# *LncRNA-ENST00000421645* Upregulates *Kank1* to Inhibit IFN-γ Expression and Promote T Cell Apoptosis in Neurosyphilis

**DOI:** 10.3389/fmicb.2021.749171

**Published:** 2021-11-30

**Authors:** Kai-Xuan Wu, Xiao-Tong Wang, Xin-Lin Hu, Xiao-Yong Jiang, Jing-Cong Zhuang, Yan-Zhu Xu, Li-Rong Lin, Man-Li Tong, Tian-Ci Yang, Li-Li Liu

**Affiliations:** ^1^Center of Clinical Laboratory, School of Medical, Zhongshan Hospital, Xiamen University, Xiamen, China; ^2^Department of Dermatology, School of Medical, Zhongshan Hospital, Xiamen University, Xiamen, China; ^3^Department of Neurology, School of Medical, Zhongshan Hospital, Xiamen University, Xiamen, China

**Keywords:** *LncRNA-ENST00000421645*, IFN-γ expression, T cell apoptosis, neurosyphilis, Kank1, *14-3-3*

## Abstract

Long non-coding RNAs are involved in many infectious diseases. Our previous studies showed that *lncRNA-ENST00000421645* expression is increased in T lymphocytes of neurosyphilis patients compared to healthy controls. However, whether *lncRNA-ENST00000421645* has biological functions remains unclear. The current study was undertaken to understand the mechanism of *lncRNA-ENST00000421645* in T lymphocyte function in neurosyphilis patients. The *lncRNA-ENST00000421645* pull-down assay showed that *lncRNA-ENST00000421645* acted on the acetylase NAT10. The chromatin immunoprecipitation (ChIP)-PCR results showed that *lncRNA-ENST00000421645* promoted the acetylation of histone H3K27 adjacent to the Kank1 promoter, thereby promoting Kank1 protein expression. Kank1 promotes 14-3-3 protein expression, inhibits NF-kB activation, inhibits IFN-γ secretion by T lymphocytes, and promotes T lymphocyte apoptosis. Taken together, our findings suggest a novel mechanism that *LncRNA-ENST00000421645* upregulates *Kank1* to inhibit IFN-γ expression and promote T cell apoptosis in neurosyphilis.

## Introduction

Neurosyphilis is a central nervous system disease caused by *Treponema pallidum* (*TP*). It is one of the most destructive and difficult clinical types of syphilis (Gonzalez et al., 2019). The mechanism of *TP*-induced neurosyphilis is not fully understood. In our previous experiments, we found an abnormal humoral immune response and cellular immune response in neurosyphilis patients (Liu et al., 2013). Assessment of cerebrospinal fluid (CSF) abnormalities in neurosyphilis patients without human immunodeficiency virus (HIV) infection indicated that CD3^+^ CD4^+^ T cells play a dominant role in the CSF of neurosyphilis patients (Leader et al., 2007; Li et al., 2013). The number of CD4^+^ T cells in the CSF of asymptomatic neurosyphilis patients and neurosyphilis patients with brain parenchymal injury was significantly higher than that in the CSF of syphilis patients without nerve injury (Leader et al., 2007). Marra et al. (1998) established a model of *TP* invasion into the nervous system and found that the T lymphocytes in the CSF were mainly CD4^+^ T cells, suggesting that T helper (Th) cells may be recruited into the nervous system to clear *TP.*

Accumulating evidence shows that lncRNA mediate the regulation of host CD4^+^ T cells and macrophages to participate in the host cell response to microbial infections (Yin et al., 2013; Yılmaz Susluer et al., 2018). For example, after *Toxoplasma gondii* infection, *non-shat022487* inhibits IL-12, tumor necrosis factor-α (TNF-α) and IFN-γ expression by downregulating the expression of the immune-activating molecule unc93b1 in human macrophages (Liu et al., 2018), contributing to the establishment of chronic *Toxoplasma* infection. In staphylococcal enterotoxin B-activated CD4^+^ T cells of C57BL/6J mice, the long non-coding RNA (lncRNA) *AW112010* inhibits IL-10 expression through histone demethylation and promotes the differentiation of inflammatory T cells (Yang et al., 2020). During *Mycobacterium tuberculosis* infection, *lncRNA-CD244* directly interacts with the polycomb repressive complexes(PRC2) subunit enhancer of zeste homolog 2(EZH2), which leads to histone H3 lysine (K) 27 methylation and confers a stronger inhibitory chromatin state at IFN-γ or TNF-α loci (Wang et al., 2015). In other words, *lncRNA-CD244* upregulates the T cell suppressor CD244 to inhibit the expression of TNF-α and IFN-γ. TNF-α expression through lncRNA may be a new mechanism of *M. tuberculosis* infection. In our previous study, the changes in the lncRNA expression profiles of CD4^+^ T lymphocytes in neurosyphilis patients and healthy controls were analyzed using a non-coding RNA microarray platform. Different lncRNA and mRNA expression profiles were found. Specifically, in the neurosyphilis group, 393 lncRNAs were significantly upregulated, 287 lncRNAs were significantly downregulated, 287 mRNAs were significantly upregulated, and 331 mRNAs were significantly downregulated in neurosyphilis patients compared with healthy controls. Gene ontology (GO) analysis showed that the enriched mRNAs had significantly related functions, such as the defense response to fungi, the defense response to bacteria, killing cells of other organisms, and destroying cells of other organisms (Liu et al., 2017). LncRNAs are tissue-specific and often target multiple mRNAs; in addition, they can be used as potential biomarkers for disease diagnosis and prognosis evaluation (Schmitt and Chang, 2016). *LncRNA-ENST00000421645* was significantly differentially expressed with a very low false discovery rate (FDR) in peripheral blood CD4^+^ T cells of neurosyphilis patients and healthy subjects. Whether the *TP*-induced expression of *lncRNA-ENST00000421645* in CD4^+^ T lymphocytes is specific remains to be studied, and the potential mechanism underlying the effect of *lncRNA-ENST00000421645* on the function of CD4^+^ T lymphocytes remains to be determined.

## Materials and Methods

### Cell Culture and Transfection Experiment

Jurkat-E6-1 human leukemia T lymphocytes (purchased from Bnbio, Inc., Beijing, China) were cultured in RPMI 1640 (Invitrogen, Carlsbad, California, United States) medium supplemented with 10% (v/v) heat-inactivated fetal bovine serum (Biological Industries Ltd., Kibbutz Beit HaEmek, Israel) at 37°C in a humidified atmosphere containing 5% CO_2_ *LncRNA-ENST00000421645* was cloned into the GV502 plasmid (GeneChem Co., Ltd., Shanghai, China). The lentiviral vector and packaging vector were transfected into the packaging cell line 293T (ATCC) with Turbofect transfection reagent (Invitrogen) in accordance with the instruction manual. The lentivirus-containing medium was thus obtained. Then, T lymphocytes were transduced with the lentiviral vectors, and the stable cells were screened with puromycin (5 μg/mL). The lentiviral *lncRNA-ENST00000421645* overexpression vector was transduced into T lymphocytes, and the resulting cells were called OE cells. The empty lentiviral vector was transduced into T lymphocytes, and the resulting cells were called NC cells. *Kank1* small interfering RNA (siRNA) was transfected into OE cells, and the resulting cells were called OEsiRNA Kank1 cells. OE cells transfected with non-targeting control siRNA were called OEsiRNA NC cells.

### CCK-8 Assay

The proliferation of T lymphocyte Jurkat-E6-1 cells, NC cells and OE cells was assessed at 24, 48, and 72 h after transfection. CCK-8 solution (10 μL; Beyotime Biotechnology Co., Ltd.) was added and incubated for 3 h, and the absorbance was measured at 450 nm as described previously (Wang et al., 2020).

### TUNEL Apoptosis Assay

Cell samples (slides) were naturally dried and immersed in 4% paraformaldehyde fixative solution for 30 min to improve the permeability of the cells. After soaking in PBS, 1% Triton X-100 was added to the samples and incubated at room temperature for 15 min. After washing, nuclease inactivation enzyme was added, and after another wash, protease K was added for cleanup. Then, 100 μL of Streptavidin-TRITC-labeled working solution was added to each sample and soaked at 37°C in the dark for 1 h. After washing, DAPI was added for re-staining, and samples were observed under a fluorescence microscope after washing.

### Real-Time qPCR Assay

The T lymphocytes were transduced with lentiviral *lncRNA-ENST00000421645* overexpression vector and empty lentiviral vector, respectively. The experiment was repeated three times to obtain three groups of OE cells and NC cells that had been transformed for 24 h. *Kank1* small interfering RNA (siRNA) was transfected into OE cells and NC cells. After transfection for 24 h, the OE cells, NC cells, OEsiRNA Kank1 and OEsiRNA NC cells were collected for RT-qPCR experiments. Total RNA was isolated from the cells using Trizol reagent (Life Technologies Corporation, Carlsbad, CA, United States). Total RNA yield and purity were analyzed spectrophotometrically (Denovix, United States) as previously reported (Chong, 2001). Then, First-strand cDNA was synthesized using a cDNA synthesis kit (Takara Biotechnology, Dalian, China) and SYBR Premix Ex Taq*™* (Takara Biotechnology, Dalian, China). Two-step real-time fluorescence quantitative PCR for *Kank1*, *14-3-3* and *IFN-*γ was used to detected the mRNA level of *Kank1*, *14-3-3* and *IFN-*γ. One microliters of each cDNA sample was amplified in a 20 μL reaction mixture containing 10 μL of SYBR premix Ex Taq II (2x) (Takara Biotechnology, Dalian, China), the forward primer and reverse primer at 0.25 μM each, and 8 μL Nuclease-Free Water in a 96-well reaction plate with a LightCycler^®^ 480 PCR-Time PCR System (Roche Diagnostics Ltd., Swit). The precise conditions for PCRs were as follows: in the pre-denaturation stage, the reaction cycle was repeated for 30 s at 95°C; at the denaturation stage, the reaction time was 5 s at 95°C, in the annealing extension phase, the reaction time was 35 s at 60°C; and the denaturation and annealing extension stage was cycled for 40 times (distilled water and negative extraction control) were included in each assay. Three biological and technical replicates were performed. The specific primers used in real-time RT-PCR analyses were listed in Table 1.

**TABLE 1:** Primer sequences were shown below.

	Forward 5′–3′	Reverse 5′–3′
*IFN-*γ	AGAGTGTGGAGA CCATCAAGGA	TGCGTTGGACAT TCAAGTCAGT
*Kank1*	AACAGGCAGCAA CACAGAGGAG	CCACAACCGAT AGACCGCACTT
*14-3-3*	TGCTGAAGTTGC GTGTGGTGAT	GCGGATTGGG TGTGTGGGTT
*GAPDH*	GAGTCAACGGA TTTGGTCGT	GACAAGCTTC CCGTTCTCAG

Total RNA was extracted from NC cells, OE cells, OEsiRNA NC cells and OEsiRNA Kank1 cells and was subjected to reverse transcription. The cDNA product of reverse transcription was used as the template in subsequent experiments. 1 μL of cDNA extracted from peripheral blood T cells among 3 clinical samples was used in this study and 4 different T cells from culture were dissolved in 9 μL water, respectively. After the mixing process, 1 μL of mixture was added into 9 μL RNA-free water, followed by the same multiple dilution twice. Standard curves of target and reference genes were established from samples which were diluted to 1 × 10^5^, 1 × 10^4^, 1 × 10^3^, 1 × 10^2^, and 1 × 10^1^. The *E*-values and corresponding *R*^2^ values were shown in Table 2. The melting curve of PCR showed that PCR amplification had specificity, with only one specific peak, no primer-dimers and no non-specific amplification products. The results showed that the slope differences of standard curves between *lncRNA-ENST00000421645* mRNA and β-actin mRNA, *IFN-*γ mRNA and *GAPDH* mRNA, *Kank1* mRNA and *GAPDH* mRNA, *14-3-3* mRNA and *GAPDH* mRNA were less than 0.1, respectively; therefore, we employed 2-ΔΔCt method (Livak and Schmittgen, 2001) to calculate the relative expression level of the target gene. The C_t_ value was adjusted automatically and the threshold cycle value difference (ΔC_t_) between FAM C_t_ of the target gene (*lncRNA- ENST00000421645, Kank1, 14-3-3*, and *IFN-*γ) and FAM C_t_ of *GAPDH* (internal control) was used to normalize the amount of *Kank1, 14-3-3* and *IFN-*γ. As long as the target gene and the internal control have similar amplification efficiencies, C_t_ values are normalized by using the difference (ΔC_t_) between the internal control and target gene. This value is calculated for each sample to be quantified. Finally, the amount of *Kank1, 14-3-3* and *IFN-*γ, normalized to an endogenous reference and relative to a calibrator, is given by:


$$\mathrm{Relative}\,\mathrm{Quantification}=2^{-\Delta \,\Delta \,\mathrm{Ct}}$$

WhereΔΔC=tΔC(Experimentalsample)t-ΔC(Controlsample)t.

Δ⁢Ct=Ct⁢(Target⁢gene)-Ct⁢(Reference⁢gene).

**TABLE 2:** The standard curve indexes of IFN-γ, Kank1,14-3-3 and GAPDH genes in four different cells were shown below.

Cell name	Gene name	E	R^2^	Tm (°C)
NC	*IFN-*γ	2.047	0.9887	82.98
NC	*Kank1*	2.062	0.9920,	81.82
NC	*14-3-3*	2.058	0.9985	87.13
NC	*GAPDH*	2.048	0.9986	84.37
OE	*IFN-*γ	1.978	0.9986	82.78
OE	*Kank1*	2.003	0.9980	81.80
OE	*14-3-3*	2.001	0.9985	87.33
OE	*GAPDH*	2.000	0.9973	84.71
OEsiRNA NC	*IFN-*γ	2.078	0.9901	82.81
OEsiRNA NC	*Kank1*	2.098	0.9878	81.71
OEsiRNA NC	*14-3-3*	2.055	0.9910	87.07
OEsiRNA NC	*GAPDH*	2.080	0.9966	84.32
OEsiRNA Kank1	*IFN-*γ	2.081	0.9921	82.65
OEsiRNA Kank1	*Kank1*	2.084	0.9912	81.90
OEsiRNA Kank1	*14-3-3*	2.061	0.9843	87.58
OEsiRNA Kank1	*GAPDH*	2.074	0.9836	84.67

*NC: T lymphocytes transfected with empty lentiviral vector, OE: T lymphocytes transfected with lncRNA-ENST00000421645 overexpression lentiviral vector, OEsiRNA NC: OE cells transfected with non-targeting control siRNA, OEsiRNA Kank1:OE cells transfected with Kank1 small interfering RNA (siRNA), E: Efficiency, R^2^: coefficient of determination, Tm: Melting Temperature.*

### ELISA

After transfection for 24 h, the IFN-γ concentration in the OE cells, NC cells, OEsiRNA Kank1 and OEsiRNA NC cells culture supernatant was determined with an Human IFN-gamma ELISABASIC kit (HRP) (Neobioscience Technology Co., Ltd., China) according to the manufacturer’s instructions. IFN-γ ELISA kits were solid-phase sandwich enzyme-linked immunosorbent assays. The kit contains a matched pair of monoclonal capture and detection antibodies, Streptavidin-HRP, ELISA standard and standard reconstitution buffer.

### Western Blot Analysis

The cells were washed with PBS for three times and then added with cell lysate RIPA (1–5 × 10^6^ cells added with 250 μL lysate). The whole process was carried out on ice. The cells were then lysed by ultrasound, and the power was adjusted to 28% for about 10 s until the cell suspension became clear. After ultrasonic lysis, the cells were placed on ice for 30 min, and then cell lysis solution was collected and centrifuged at 13,200 rpm at 4°C for 30 min. The supernatant was carefully absorbed (be careful not to remove precipitation) into a clean 1.5 mL EP tube. 5 × loading buffer, 0.25 times of supernatant volume, was added and fully mixed, heated at 100^°^C for 10 min and stored at −20^°^C for later use. The membrane transfer process begins immediately after electrophoresis. Then the membrane was incubated first with anti-14-3-3, anti-kank1, anti-p-ikba, anti-t-ikba (all 1:1,000; Abcam, Cambridge, Ma, United States) and anti-GAPDH (1:1,000, Cell Signaling Technology, Danvers, MA, United States) antibodies and then with horseradish peroxidase-conjugated secondary antibodies. Western Chemiluminescent HRP 149 Substrate (ECL, Millipore, United States) was used to visualize the immunoreactivity bands. The images were quantified using ImageJ software.

### Chromatin Immunoprecipitation-PCR Assay

ChIP was used to investigate the binding of histone H3K27 to the target gene *Kank1* in NC cells and OE cells. After 48 h of culture, the chromatin in the cells was first cross-linked using 1% formaldehyde, and the cells were subjected to fixation followed by lysis and enzymatic digestion. Immunoprecipitation was then conducted. The IgG ChIP antibody (2 μg, Cell Signaling Technology, Danvers, MA, United States) and the anti-H3K27 ChIP antibody (2 μg, Cambridge, Ma, United States) (Liu et al., 2021) were added. The immunoprecipitation reaction was performed for 2 h; samples were incubated on a shaking platform at 4°C overnight. After elution of the immunoprecipitates, the DNA was purified. Enrichment of histone H3K27 and the target gene *Kank1* was detected by RT-qPCR. All samples were tested in triplicate, and three multiple holes were made in each experiment.

### RNA Pull-Down Assay

Streptavidin-conjugated magnetic beads were used to pull down *lncRNA-ENST00000421645* according to the manual for the Pierce*™* Magnetic RNA-Protein pull-down kit (Thermo Fisher Scientific, United States). Biotinylated RNA and T lymphocyte lysates were mixed and incubated. Streptavidin-agarose beads were added to each binding reaction, and the mixtures were incubated at 4°C for 1 h prior to two elution steps with detergent solution. The eluted proteins were detected by mass spectrometry (MS).

### Statistical Analysis

Continuous variables are presented in the form of mean ± standard deviation. Non-normal distribution parameters are described by median ± interquartile range. The expression level of *14-3-3* mRNA level was analyzed by using rank sum test. The multiple comparison of *14-3-3* expression level was conducted using Kruskal-Wallis test. The apoptosis rates of OEsiRNA NC cells and OEsiRNA, and the mRNA level and protein level of these two cell lines were analyzed by using Student’s *t*-test. One-way ANOVA was employed to compare the mRNA levels that measured from more than two groups, and SNK test was used for conducting multiple comparisons. All statistical analyses were performed in SPSS version 20 and a *P*-value less than 0.05 was considered significant.

### Ethics Approval Statement

This study was approved by the Ethics Committee of Zhongshan Hospital (NO. 2021-147) and was in accordance with the Helsinki Declaration. Participants signed written informed consent prior to participating in the study.

## Results

### *LncRNA-ENST00000421645* Mediates Apoptosis in T Lymphocytes and Inhibited the Secretion of IFN-γ

**FIGURE 1:**
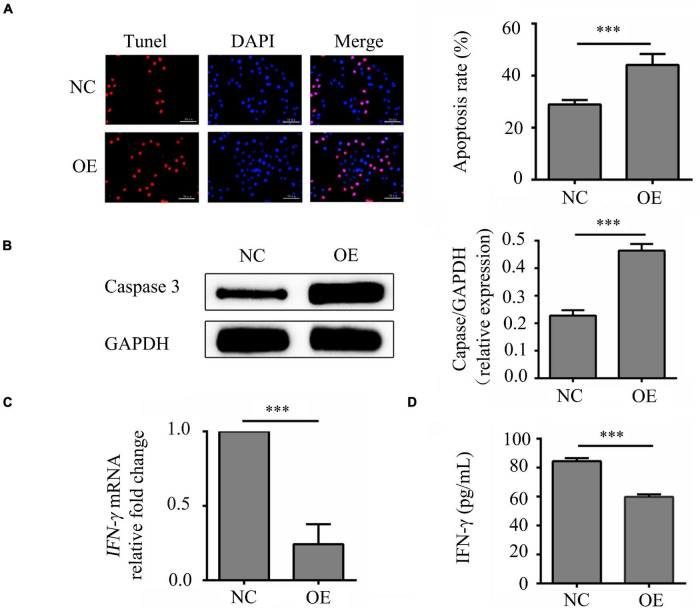
Effects of *lncRNA-ENST00000421645* on apoptosis and secretion of IFN-γ in T lymphocytes. **(A)** Apoptosis was detected in T lymphocytes transfected with empty lentiviral vector (NC cells) and T lymphocytes transfected with *lncRNA-ENST00000421645* overexpression lentiviral vector (OE cells) with a TUNEL apoptosis detection kit. **(B)** The protein level of caspase3 in NC cells and OE cells was determined by Western blot analysis. **(C)** The relative fold change in *IFN-*γ mRNA expression in NC cells and OE cells was determined by RT-qPCR. **(D)** The IFN-γ protein concentration in NC cells and OE cells was measured by ELISA (^***^P < 0.001). Student’s *t*-test was used for comparisons between two groups. Two-tailed *P-*values less than 0.05 were considered statistically significant.

In order to study on the mechanistic of *lncRNA-ENST00000421645* based on our previous study (Liu et al., 2017), the vector GV502 was used to construct the lentiviral *lncRNA-ENST00000421645* overexpression vector. T lymphocytes were transduced with lentiviral vectors. After transduction for 24 h, the apoptosis rates of NC cells OE cells were 28.91 ± 3.58% and 44.12 ± 5.24%, respectively. The apoptosis rate of OE cells was significantly increased compared with that of NC cells (*P* < 0.001, Figure 1A). Caspase3 is closely related to cell apoptosis. To further confirm the effect of *lncRNA-ENST00000421645* on T lymphocyte apoptosis, the expression of caspase3 in NC cells and OE cells was analyzed by Western blotting after transduction for 24 h. The relative caspase3 protein level in NC cells was found to be 0.228 ± 0.019. The relative caspase3 protein expression level in OE cells was 0.464 ± 0.024, indicating significant upregulation (*P* < 0.001, Figure 1B). These results suggested that overexpression of *lncRNA-ENST00000421645* can promote apoptosis in T lymphocytes.

The samples of *IFN-*γ had a similar PCR efficiency to samples containing *GAPDH*. The relative fold change in *IFN-*γ mRNA expression in OE cells compared to NC cells was 0.23, a significant decrease (*P* < 0.001, Figure 1C). In addition, the ELISA results were consistent with the RT-qPCR results. By ELISA, the IFN-γ protein concentration was determined to be 84.47 ± 7.63 pg/mL in NC cells and 59.77 ± 7.65 pg/mL in OE cells. Thus, compared with that in NC cells, the content of IFN-γ in OE cells was significantly reduced (*P* < 0.001, Figure 1D). These results showed that *lncRNA-ENST00000421645* inhibits the secretion of IFN-γ.

### *LncRNA-ENST00000421645* Affects the Expression of the Adjacent Gene *Kank1*

**FIGURE 2:**
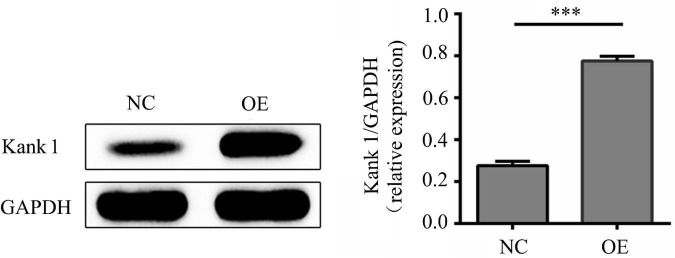
The protein level of Kank1 in NC cells and OE cells was determined by Western blot analysis. The relative protein expression level of Kank1 in OE cells was significantly increased compared to that in NC cells (77.33 ± 2.08% vs. 27.67 ± 2.08%, ***P < 0.001). Student’s *t*-test was used for comparisons between two groups. Two-tailed *P*-values less than 0.05 were considered statistically significant.

According to lncRNA expression profile analysis, the gene adjacent to *lncRNA-ENST00000421645* is *Kank1*. The expression of Kank1 in OE cells was analyzed by Western blotting, and the relative Kank1 protein expression level in NC cells was found to be 0.277 ± 0.021. The relative Kank1 protein expression level in OE cells was 0.773 ± 0.021, indicating significant upregulation (*P* < 0.001, Figure 2).

### *LncRNA-ENST00000421645*-Regulated Acetylation of Histone H3K27 Adjacent to the *Kank1* Promoter Promotes the Expression of *Kank1*

Bioinformatics showed that the adjacent gene of *lncRNA-ENST00000421645* is Kank1 according to our previous published paper (Liu et al., 2017). To investigate the mechanism by which *lncRNA-ENST00000421645* upregulates the expression of the adjacent gene *Kank1*, a *lncRNA-ENST00000421645* pull-down assay was performed with streptavidin-conjugated magnetic beads. The precipitated product was analyzed by mass spectrometry to identify the *lncRNA-ENST00000421645* interacting proteins. A total of 793 proteins were identified in the sense group, and 638 proteins were identified in the anti-sense group. Mass spectrometry analysis of the precipitated products revealed 155 differential proteins. The top ten differential proteins were listed in Table 3. The changes in chromatin structure caused by the modification of histones play an important role in the regulation of gene expression and transcription. Among them, histone acetylation is particularly important. The acetylation of the N-terminal lysine residues of histones in eukaryotic cells is related to the transcriptional activation of genes. We analyzed that among the top 10 proteins that bind to *lncRNA-ENST00000421645*, there is a protein named NAT10 related to acetylation. The acetylase NAT10 ranked ninth, indicating that *lncRNA-ENST00000421645* acted on this protein.

**TABLE 3:** Top fifteen differential proteins were listed below.

Sequential number	Peptides	Unique peptides	Group description
1	19	7	DNA topoisomerase 2-beta OS = Homo sapiens GN = TOP2B PE = 1 SV = 3
2	10	6	CLIP-associating protein 2 OS = Homo sapiens GN = CLASP2 PE = 1 SV = 1
3	5	1	Y-box-binding protein 3 OS = Homo sapiens GN = YBX3 PE = 1 SV = 4
4	7	7	AT-rich interactive domain-containing protein 1A OS = Homo sapiens GN = ARID1A PE = 1 SV = 2
5	7	7	AT-rich interactive domain-containing protein 1A (Fragment) OS = Homo sapiens GN = ARID1A PE = 1 SV = 1
6	7	7	AT-rich interactive domain-containing protein 1A OS = Homo sapiens GN = ARID1A PE = 1 SV = 3
7	9	6	ATP-dependent RNA helicase DDX3Y OS = Homo sapiens GN = DDX3Y PE = 1 SV = 2
8	6	6	Ubiquitin-associated protein 2-like OS = Homo sapiens GN = UBAP2L PE = 1 SV = 2
9	9	9	RNA cytidine acetyltransferase OS = Homo sapiens GN = NAT10 PE = 1 SV = 2
10	5	2	Polyadenylate-binding protein OS = Homo sapiens GN = PABPC4 PE = 1 SV = 1

**FIGURE 3:**
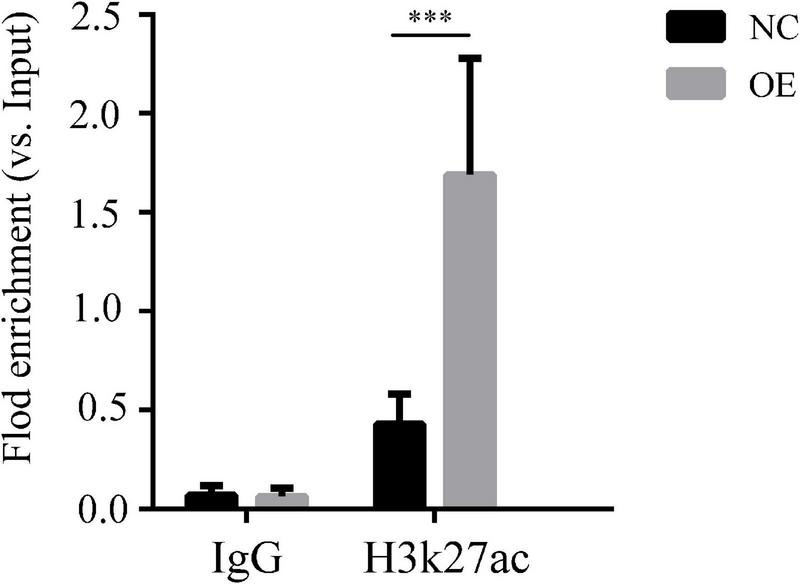
Acetylation of histone H3K27 adjacent to the Kank1 promoter was detected by ChIP. All samples were tested in triplicate, and three multiple holes were made in each experiment. Enrichment of the target gene *Kank1* was detected by RT-qPCR (^***^P < 0.001). Student’s *t*-test was used for comparisons between two groups. Two-tailed *P*-values less than 0.05 were considered statistically significant.

We verified that *lncRNA-ENST00000421645* may promote the acetylation of histone H3K27 adjacent to the *Kank1* promoter through the acetylase NAT10, thus promoting the expression of Kank1. ChIP-PCR was performed on OE cells and NC cells. An anti-H3K27 antibody was used to precipitate the cross-linked DNA-protein complexes. Primers targeting the *Kank1* promoter sequence were designed, and the DNA sequence enriched by the anti-H3K27 antibody was detected by real-time PCR. The samples of *Kank1* had a similar PCR efficiency to samples containing *GAPDH*. The enrichment of the *Kank1* gene promoter in the non-H3K27-acetylated group of OE cells was significantly increased 3.96-fold compared with that in NC cells (*P* < 0.001, Figure 3). These results suggest that *lncRNA-ENST00000421645* promotes the acetylation of histone H3K27 adjacent to the *Kank1* promoter, thereby promoting Kank1 expression. All samples were tested in triplicate.

### *LncRNA-ENST00000421645* Promotes Kank1 and 14-3-3 Protein Expression and Inhibits NF-kB Pathway Activation

**FIGURE 4:**
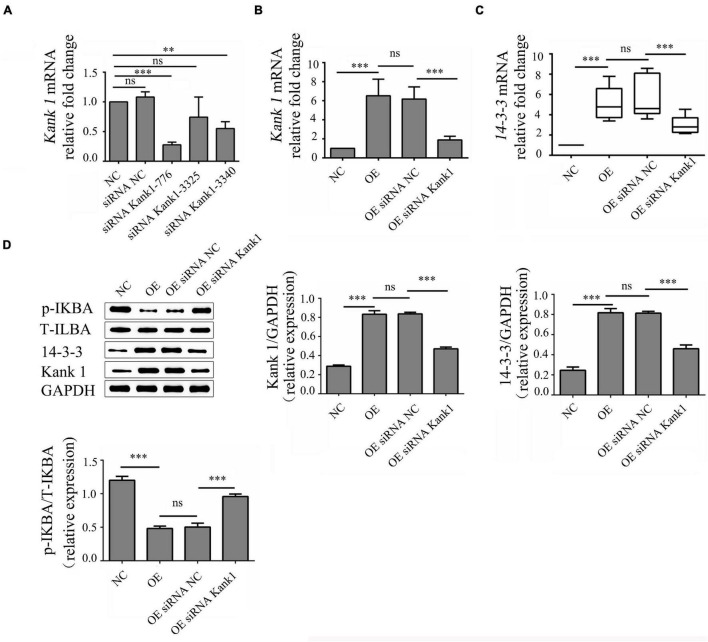
*LncRNA-ENST00000421645* promoted the protein expression of Kank1 and 14-3-3 and inhibited NF-kB pathway activation. **(A)** The *Kank1* gene knockdown efficiency of dKank1 siRNA in T lymphocytes was evaluated by RT-qPCR, and the siRNA with the highest efficiency—*Kank1-776*—was selected. **(B)** The relative mRNA level of *Kank1* in NC cells, OE cells, OEsiRNA NC cells, and OEsiRNA Kank1 cells was determined by RT-qPCR. **(C)** The relative mRNA level of *14-3-3* in NC cells, OE cells, OEsiRNA NC cells, and OEsiRNA Kank1 cells was determined by RT-qPCR. **(D)** The protein levels of p-IKBA, T-IKBA, 14-3-3 and Kank1 in NC cells, OE cells, OEsiRNA NC cells and OEsiRNA Kank1 cells were determined by Western blot analysis (^**^P < 0.01, ^***^P < 0.001). *14-3-3* mRNA level was analyzed by using rank sum test. The multiple comparison of *14-3-3* expression level was conducted using Kruskal-Wallis test. *Kank1* mRNA and protein level of p-IKBA, T-IKBA, 14-3-3 and Kank1 were analyzed by using One-way ANOVA, and SNK test was used for conducting multiple comparison. *P*-values less than 0.05 were considered statistically significant.

Kank1 siRNA was used to knock down *Kank1* gene expression in OE cells, and RT-qPCR was then used to detect the knockdown efficiency and select the siRNA with the highest efficiency—Kank1-776—for subsequent experiments (*P* < 0.001, Figure 4A). The relative fold change in the *Kank1* mRNA level in OE cells compared with NC cells was 6.52 ± 1.75, a significant increase compared with the level in NC cells (*P* < 0.001, Figure 4B). The relative fold change in the *Kank1* mRNA level in OEsiRNA NC cells compared with OE cells was 6.18 ± 1.27, a difference that was not significant. The relative fold change in the *Kank1* mRNA level in OEsiRNA Kank1 cells was 1.89 ± 0.38, a significant reduction compared with that in OEsiRNA NC cells (*P* < 0.01, Figure 4B). Kank 1 has also been shown to promote 14-3-3 protein expression to inhibit NF-kB activation (Kakinuma et al., 2008). The samples of *14-3-3* had a similar PCR efficiency to samples containing *GAPDH*. The relative fold change in the *14-3-3* mRNA level in OE cells was 4.88 ± 0.58, a significant increase compared with that in NC cells (*P* < 0.001, Figure 4C). The relative fold change in the *14-3-3* mRNA level in OEsiRNA-NC cells compared with OE cells was 5.43 ± 0.59, a difference that was not significant. The relative fold change in the *14-3-3* mRNA level in OEsiRNA Kank1 cells was 2.93 ± 0.40, a significant reduction compared to that in OEsiRNA NC cells (*P* < 0.01, Figure 4C). In addition, the Western blot results were consistent with the RT-qPCR results. The relative Kank1 protein levels in NC cells, OE cells, OEsiRNA NC cells and OEsiRNA Kank1 cells were 0.288 ± 0.013, 0.833 ± 0.037, 0.837 ± 0.017, and 0.471 ± 0.018, respectively. The Kank1 protein level in OEsiRNA Kank1 cells was significantly lower than that in OEsiRNA NC cells (*P* < 0.001, Figure 4D). The relative 14-3-3 protein levels in NC cells, OE cells, OEsiRNA NC cells and OEsiRNA Kank1 cells were 0.246 ± 0.033, 0.817 ± 0.043, 0.813 ± 0.018, and 0.460 ± 0.037, respectively. The relative 14-3-3 protein expression level was also reduced in OEsiRNA Kank1 cells compared with OEsiRNA NC cells (*P* < 0.001, Figure 4D).

The phosphorylation level of IKBa can indirectly reflect the activation of the NF-kB signaling pathway. P-IKBa/T-IKBa refers to the ratio of phosphorylated IKBa protein to total IKBa protein content, which can reflect the activation degree of NF-kB pathway. Thus, the phosphorylation level of IKBa was analyzed by Western blotting. The relative p-IKBa/IKBa ratios in NC cells, OE cells, OEsiRNA NC cells and OEsiRNA Kank1 cells were 1.200 ± 0.058, 0.482 ± 0.035, 0.503 ± 0.058, and 0.958 ± 0.038, respectively. Compared with that in NC cells, the relative p-IKBa/IKBa ratio was significantly reduced in OE cells (*P* < 0.001; Figure 4D). Compared with that in OEsiRNA NC cells, the relative p-IKBa/IKBa ratio was obviously increased in OEsiRNA Kank1 cells (*P* < 0.001, Figure 4D).

This analysis showed that *lncRNA-ENST00000421645* upregulated the expression of Kank1. Kank1 promoted 14-3-3 protein expression and inhibited NF-kB activation.

### *LncRNA-ENST00000421645* Promotes Apoptosis and Inhibits IFN-γ Secretion Through Kank1 in T Lymphocytes

**FIGURE 5:**
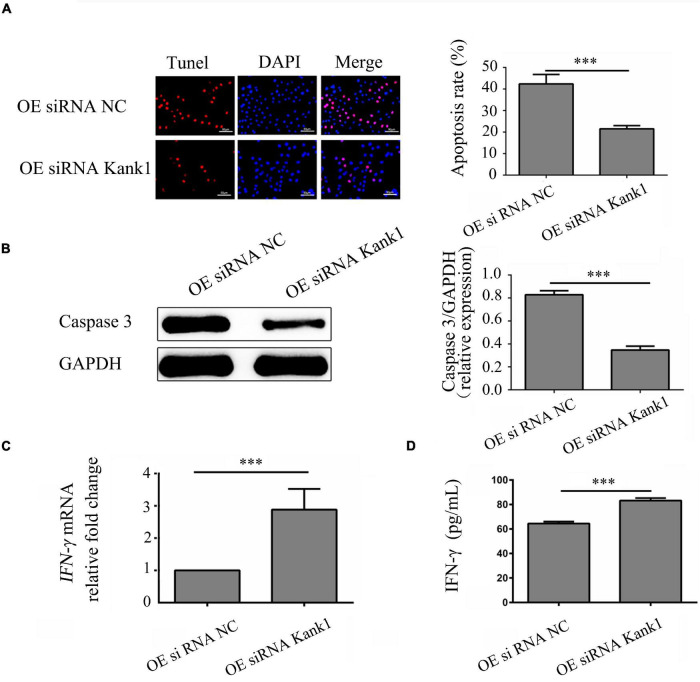
*LncRNA-ENST00000421645* promoted apoptosis and inhibited the secretion of IFN-γ through Kank1 in T lymphocytes. **(A)** Apoptosis was detected in OEsiRNA NC cells and OEsiRNA Kank1 cells with a TUNEL apoptosis detection kit. **(B)** The protein level of caspase3 in OEsiRNA NC cells and OEsiRNA Kank1 cells was determined by Western blot analysis. **(C)** The relative fold change in *IFN-*γ mRNA expression in OEsiRNA NC cells and OEsiRNA Kank1 cells was determined by RT-qPCR. **(D)** The IFN-γ protein concentration in OEsiRNA NC cells and OEsiRNA Kank1 cells was measured by ELISA (^***^P < 0.001). Student’s *t*-test was used for comparisons between two groups. Two-tailed *P*-values less than 0.05 were considered statistically significant.

**FIGURE 6:**
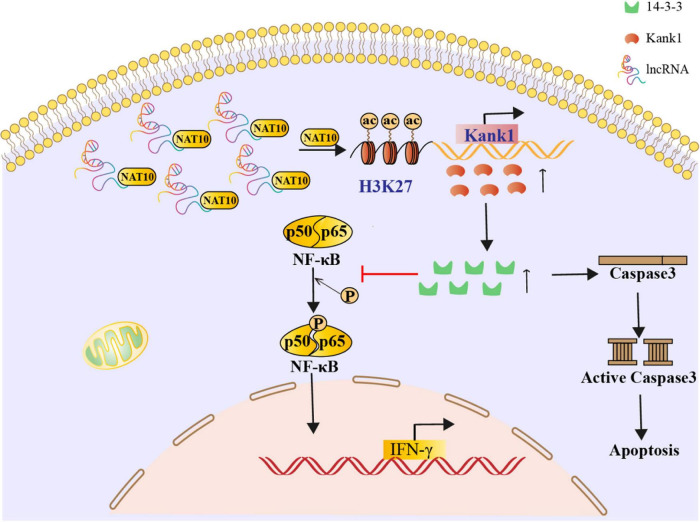
A mechanistic diagram of T lymphocyte regulation by *lncRNA-ENST00000421645* was shown.

The effect of the *lncRNA-ENST00000421645*–Kank1 protein interaction on T lymphocyte function was further investigated. The apoptosis rates of OEsiRNA NC cells and OEsiRNA Kank1 cells were 42.35 ± 4.16% and 21.50 ± 3.81%, respectively. Compared with that of OEsiRNA NC cells, the apoptosis rate of OEsiRNA Kank1 cells was significantly reduced (*P* < 0.001, Figure 5A). To further confirm the effect of Kank1 on T lymphocyte apoptosis, after transfection for 24 h, the expression of caspase3 in OE siRNA NC cells and OE siRNA Kank1 cells was analyzed by Western blotting. The relative caspase-3 protein expression level was significantly decreased in OE siRNA Kank1 cells compared with OE siRNA NC cells (0.347 ± 0.034 vs. 0.829 ± 0.035, *P* < 0.001; Figure 5B). The relative fold change in the *IFN-*γ mRNA level in OE siRNA Kank1 cells compared with OEsiRNA NC cells was 2.88 ± 0.65, a significant increase (*P* < 0.001, Figure 5C). In addition, the ELISA results were consistent with the RT-qPCR results. The IFN-γ protein levels in OEsiRNA Kank1 and OEsiRNA NC cells were 83.20 ± 4.86 pg/mL and 64.42 ± 5.30 pg/mL, respectively. Compared with that in OE siRNA NC cells, the IFN-γ level in OE siRNA Kank1 cells was obviously increased (*P* < 0.001, Figure 5D). These results indicated that *lncRNA-ENST00000421645* was overexpressed in T lymphocytes of neurosyphilis patients, resulting in promotion of apoptosis and inhibition of IFN-γ secretion through the Kank1 protein. The mechanistic diagram of T lymphocyte regulation by *lncRNA-ENST00000421645* was shown in Figure 6.

## Discussion

Previous studies have shown that many lncRNAs are involved in regulating the immune response in response to pathogen invasion (Jiang et al., 2018). Li et al. (2016) found that the lncRNA *NRON* in CD4^+^ T cells can specifically downregulate Tat protein expression and is beneficial to latent HIV-1 virus *in vivo*. Yao et al. found that *Helicobacter pylori* infection can activate the SGK1/JunB signaling pathway to enhance the expression of the lncRNA *LNC-SGK1* and induce an increase in CD4^+^ T cell differentiation into Th2 and Th17 cells and a decrease in differentiation into Th1 cells (Yao et al., 2016). Serum *Lnc-SGK1* expression is associated with poor prognosis in patients with gastric cancer. Our previous study suggests increased expression of *lncRNA-ENST00000421645* was found in CD4^+^ T cells from neurosyphilis patients compared to healthy controls (Liu et al., 2017), but its function is unclear.

Recently, researchers have begun to study the function of lncRNAs and have found that lncRNAs can affect cell proliferation, migration, differentiation, apoptosis, and other physiological processes (Jou et al., 2019; Wang et al., 2019; Xie et al., 2019). In this study, we demonstrated that *lncRNA-ENST00000421645* can promote apoptosis in T lymphocytes and inhibit IFN-γ secretion. *LncRNA-ENST00000421645* was overexpressed in a T lymphocyte line (Jurkat-E6-1), and *lncRNA-ENST00000421645* was found to promote the expression of the apoptosis-related protein caspase3 in T lymphocytes and promote T lymphocyte apoptosis. Caspase3 is a major effector caspase and is a common downstream effector molecule of multiple apoptotic pathways that plays a central role in the process of apoptosis; thus, it is called a “death executing protease (Li et al., 1997; Porter and Jänicke, 1999; Winstel et al., 2019; Soleimani and Sajedi, 2020). Our findings suggested that *lncRNA-ENST00000421645* induced apoptosis in T lymphocytes through the caspase pathway.

To explore the mechanism by which *lncRNA-ENST00000421645* promotes T lymphocyte apoptosis and inhibits IFN-γ secretion, a series of additional experiments were carried out. Our previous experiments showed through nuclear separation technology that *lncRNA-ENST00000421645* exists mainly in the cytoplasm (results not shown). Cytoplasmic lncRNAs play a role in post-transcriptional regulation mainly by regulating mRNA degradation and protein translation (Nance et al., 2020). Previous bioinformatic analysis showed that *Kank1* was an adjacent gene of *lncRNA-ENST00000421645*. In addition, *Kank1* is a documented tumor suppressor gene that inhibits the proliferation, migration, and invasion of tumor cells (gastric cancer, lung cancer, etc.) and promotes their apoptosis. This study demonstrated that *lncRNA-ENST00000421645* can upregulate the expression of the neighboring gene *Kank1*. The interaction between *lncRNA-ENST00000421645* and NAT10 was proven by RNA immunoprecipitation with mass spectrometry. NAT10 is a histone acetyltransferase and promotes histone acetylation and chromosome depolymerization, thus promoting transcription and translation. In this study, ChIP-PCR showed that *lncRNA-ENST00000421645* promoted the acetylation of histone H3K27 adjacent to the *Kank1* promoter and thus promoted Kank1 expression. Increased Histone acetylation can loosens the chromatin structure and facilitates transcription.

We further explored the mechanism of *lncRNA-ENST00000421645* through upregulation of *Kank1*. In this study, *Kank1* gene expression was knocked down in OE cells. When Kank1 expression was knocked down, caspase3 expression was downregulated, indicating that *lncRNA-ENST00000421645* promoted apoptosis in T lymphocytes by upregulating Kank1 protein expression.

Kank1 has also been shown to promote 14-3-3 protein expression to inhibit NF-kB activation (Kakinuma et al., 2008). Although the 14-3-3 protein has no intrinsic protease activity, it has a wide range of biological functions. It participates in regulating cell signal transduction, cell metabolism, cell invasion, and many other cellular physiological activities. NF-kB is an important transcriptional regulator in the nucleus that can promote cell proliferation and inhibit apoptosis. Aguilera et al. (2006) reported that 14-3-3 regulates the NF-kB signaling pathway by binding to NF-kB p65 and IKBα. We further investigated whether the upregulated *lncRNA-ENST00000421645* in CD4^+^ cells of neurosyphilis patients regulates Kank1 and thus plays an important role in 14-3-3 protein expression and NF-kB pathway activation. In this study, after *Kank1* gene expression was knocked down in T lymphocytes by siRNA, the *14-3-3* gene and 14-3-3 protein expression levels were significantly decreased and the p-IKBA level was increased in T lymphocytes with *Kank1* knockdown. This finding demonstrated that *lncRNA-ENST00000421645* promoted 14-3-3 protein expression through Kank1 and then inhibited NF-kB signaling pathway activation, thereby suppressing IFN-γ secretion. IFN-γ plays an important role in the activation, growth, and differentiation of T cells and is indispensable for inflammatory and cell-mediated immune responses (Ushio et al., 1996). Interactions between lncRNAs and IFN-γ loci control the expression of IFN-γ. The first lncRNA identified to regulate IFN-γ expression was *NeST*. *NeST*, with a length of 170 KB, is located downstream of the *IFN-*γ gene and interacts with IFN-γ as an anti-sense lncRNA (Vigneau et al., 2003). *NeST* binds to WDR5 to activate H3K4 and drive methylation to enhance chromatin accessibility, which in turn reduces the recruitment of local histones at nearby IFN-γ loci and promotes IFN-γ transcription (Vigneau et al., 2003). The lncRNA *NRON* sequesters *NFAT* in the cytoplasm, inhibiting it, and inhibits the interaction between *NFAT* and the *IFN-*γ promoter, thus suppressing the expression of IFN-γ (Willingham et al., 2005). The lncRNA *SROS1* inhibits the binding of *STAT1* mRNA to the RBP CAPRIN1, thereby disrupting *STAT1* mRNA stability and thereby inhibiting IFN-γ-mediated clearance of *Listeria monocytogenes* from macrophages. As discussed by Willingham et al. (2005) and Booty et al. (2016) when the expression of IFN-γ is low, susceptibility to mycobacterial and fungal infections is common. Does low expression of IFN-γ also increase susceptibility to *TP* infection*?* We speculated that the high expression of *lncRNA-ENST00000421645* in T lymphocytes of neurosyphilis patients accelerated the apoptosis of CD4^+^ T cells and thus impaired the immune response of T lymphocytes. Inhibition of IFN-γ expression contributes to the immune escape of *TP*, which survives in the nervous system, eventually leading to the occurrence of neurosyphilis. Interventions that inhibit T lymphocyte apoptosis and suppress the expression of *lncRNA-ENST00000421645* may constitute a novel therapeutic strategy to reduce the occurrence of neurosyphilis in syphilis patients.

The limitations were that the result of *lncRNA-ENST00000421645* promoting the acetylation of histone H3K27 adjacent to the Kank1 promoter would be better if there is a WB map. The underlying mechanism of Kank1 driving the expression of 14-3-3 is not clear and requires further studies. However, this study filled in the knowledge gap regarding the function of *lncRNA-ENST00000421645* and clarified a new mechanism of the transcriptional regulation of IFN-γ by *lncRNA-ENST00000421645*.

## Data Availability Statement

The original contributions presented in the study are included in the article/supplementary material, further inquiries can be directed to the corresponding author/s.

## Ethics Statement

The studies involving human participants were reviewed and approved by the Ethics Committee of Zhongshan Hospital and was in accordance with the Helsinki Declaration. The patients/participants provided their written informed consent to participate in this study.

## Author Contributions

K-XW, X-TW, and L-LL conceived and designed the experiments. K-XW, X-TW, and M-LT performed the experiments. X-LH, X-YJ, and J-CZ analyzed the data. Y-ZX, L-RL, and T-CY contributed reagents, materials, and analysis tools. K-XW and L-LL wrote the manuscript. All authors contributed to the article and approved the submitted version.

## Conflict of Interest

The authors declare that the research was conducted in the absence of any commercial or financial relationships that could be construed as a potential conflict of interest.

## Publisher’s Note

All claims expressed in this article are solely those of the authors and do not necessarily represent those of their affiliated organizations, or those of the publisher, the editors and the reviewers. Any product that may be evaluated in this article, or claim that may be made by its manufacturer, is not guaranteed or endorsed by the publisher.
